# Characterizing a Unique Retinal Phenotype in *INTS11*-Associated Neurodevelopmental Disorder

**DOI:** 10.1167/iovs.67.3.21

**Published:** 2026-03-11

**Authors:** Siying Lin, Wendy D. Tan, Anthony G. Robson, Gavin Arno, Paul Gissen, Elena R. Schiff, Brian McMillan, J. Vernon Odom, Omar A. Mahroo, Andrew R. Webster, Monique Leys

**Affiliations:** 1 Division of Evolution, Infection and Genomics, School of Biological Sciences, Faculty of Biology, Medicine and Health, University of Manchester, Manchester, United Kingdom; 2 Manchester Centre for Genomic Medicine, Saint Mary's Hospital, Manchester University NHS Foundation Trust, Manchester, United Kingdom; 3 Moorfields Eye Hospital, London, United Kingdom; 4 UCL Institute of Ophthalmology, University College London, London, United Kingdom; 5 Department of Ophthalmology and Visual Sciences, West Virginia University, Morgantown, West Virginia, United States; 6 Division of Research, Greenwood Genetic Center, Greenwood, South Carolina, United States; 7 Department of Paediatric Metabolic Medicine, Great Ormond Street Hospital for Children NHS Trust, London, United Kingdom; 8 University College London Great Ormond Street Institute of Child Health, London, United Kingdom; 9 National Institute of Health Research Great Ormond Street Biomedical Research Centre, London, United Kingdom; 10 Department of Ophthalmology, St Thomas’ Hospital, London, United Kingdom

**Keywords:** *INTS11*, retina, neurodevelopmental disease, retinal dystrophy, electronegative ERG

## Abstract

**Purpose:**

To characterize the retinal phenotype associated with *INTS11*-related neurodevelopmental disorder, expanding the phenotypic and genotypic spectrum of this newly described condition.

**Methods:**

Four affected individuals with biallelic *INTS11* variants from two unrelated families were evaluated through comprehensive ophthalmic and systemic clinical assessments, including multimodal retinal imaging and electrophysiology. Genetic testing involved genome or exome sequencing and segregation analysis, with novel variants assessed for pathogenicity. Two individuals (A-1 and A-2) had been reported previously and were noted to have retinal dystrophy, but no retinal imaging or electrophysiological findings were described; this study provides the first detailed characterization of their ocular phenotype.

**Results:**

All four affected individuals exhibited a neurodevelopmental phenotype consistent with *INTS11*-associated disease. A similar retinal phenotype was observed across all four patients. Fundus examination demonstrated mild optic disc pallor; retinal pseudocolor and autofluorescence imaging were otherwise unremarkable. Optical coherence tomography revealed severe thinning of the inner retinal layers with preserved outer retinal layers. Electroretinography demonstrated generalized rod and cone system dysfunction localized to the inner retina or post-phototransduction. Individuals A-1 and A-2 harbored biallelic missense *INTS11* variants c.34G > A; p.(Gly12Ser) and c.1219C > T; p.(Pro407Ser), as previously described. Novel missense *INTS11* variants (c.721G > A, p.(Ala241Thr) and c.983T > A, p.(Leu328Gln)) were identified in individuals B-3 and B-4.

**Conclusions:**

This study consolidates retinopathy as a feature of *INTS11*-associated neurodevelopmental disorders and provides a detailed characterization of a distinctive retinal phenotype. While most monogenic retinopathies affect the outer retina, this disease leads to thinning of the inner retinal layers. The findings underscore the importance of comprehensive ophthalmic evaluations in such cases. Furthermore, the study expands both the phenotypic and genotypic spectrum of *INTS11*-associated disorders.

The Integrator complex is a large multi-subunit complex that associates with the C-terminal domain of RNA polymerase II for 3′-end processing of a variety of nascent noncoding RNAs in the nucleus, such as small nuclear RNA (snRNA), enhancer RNA, telomerase RNA, and even several herpesvirus RNA transcripts.^1^^–^^4^ In addition, the complex also plays a broader role in transcriptional regulation of protein-coding genes at promoters, where it is involved in both the initiation of transcription and the release of RNA polymerase II from promoter-proximal pausing.^3^ The complex is composed of 15 Integrator subunits (INTS1–15), which hold various catalytic or structural functions.^5^ The *INTS11* (Integrator subunit 11) gene (MIM *611354), also known as *CPSF3L* (cleavage and polyadenylation specificity factor 3-like), encodes for the INTS11 Integrator subunit that plays an integral role in the enzymatic activity of the Integrator complex, as it possesses metallo-β-lactamase/β-CASP family RNA endonuclease activity responsible for the Integrator complex's nascent RNA processing.^6^ INTS11 works in association with INTS4 and INTS9 for RNA processing, which together are referred to as the Integrator cleavage module in the complex.^7^

Biallelic variants in *INTS11* have recently been linked to a neurodevelopmental disorder in 15 individuals from 10 unrelated families. These individuals presented with developmental delay, intellectual disability, language delay, and motor deficits. The majority also exhibited microcephaly and hypotonia, with some incidence of seizures and facial dysmorphisms noted. Ophthalmic abnormalities were observed in a subset of patients, including optic atrophy in five affected individuals and retinal dystrophy in two affected siblings.^8^ Pathogenic variants in additional Integrator subunits have also been associated with ocular involvement, including cataract, coloboma, and optic atrophy in *INTS1*-, *INTS8*-, *INTS13*-, and *INTS15*-related disorders.^9^^–^^11^ These reports suggest that disruption of the Integrator complex can affect ocular development and function, supporting a broader link between Integrator biology and eye phenotypes.

This study presents detailed ophthalmic imaging and electrophysiology findings for the two siblings with retinal dystrophy initially described above (Tepe et al.,^8^ family 8, subjects 10 and 11; family A in this study), as well as for two additional siblings from an unrelated family with *INTS11*-associated neurodevelopmental syndrome (family B). This work characterizes the unique retinopathy observed in these four individuals, offering new insights into the retinal manifestations of *INTS11*-related disorders.

## Methods

This study adhered to the Declaration of Helsinki and received relevant local research ethics approval (Moorfields Eye Hospital and the Northwest London Research Ethics Committee; 12/LO/0141 and West Virginia University Institutional Review Board, Protocol 2406992409). Written informed consent was obtained. Comprehensive clinical histories and in-depth phenotyping, including ophthalmic and systemic assessments, were conducted. Ophthalmic imaging included ultra-widefield pseudocolor and autofluorescence imaging (Optos plc, Dunfermline, UK) and spectral-domain optical coherence tomography (SD-OCT; Heidelberg Spectralis, Heidelberg, Germany).

Visual electrophysiology included dark-adapted (DA) and light-adapted (LA) full-field electroretinogram (ERG) and pattern electroretinogram testing, incorporating or based on the International Society for Clinical Electrophysiology of Vision (ISCEV) recommended or standard methods.^12^^,^^13^ Recordings were obtained using silver thread corneal electrodes using Diagnosys systems (Diagnosys LLC, Lowell, MA, USA). One individual underwent additional DA red flash ERG testing,^14^ which forms part of the routine ERG protocol at the testing institution.

Individuals A-1 and A-2 underwent whole-genome sequencing through the UK 100,000 Genomes Project,^15^^,^^16^ with variant prioritization as previously described.^8^ Individuals B-3 and B-4 underwent clinical exome sequencing with mitochondrial genome sequencing and deletion testing with GeneDx (Gaithersburg, MD, USA).

All identified variants were classified according to guidelines established by the American College of Medical Genetics & Genomics^17^ and the UK-based Association of Clinical Genomic Science.^18^

## Results

**Figure 1.**
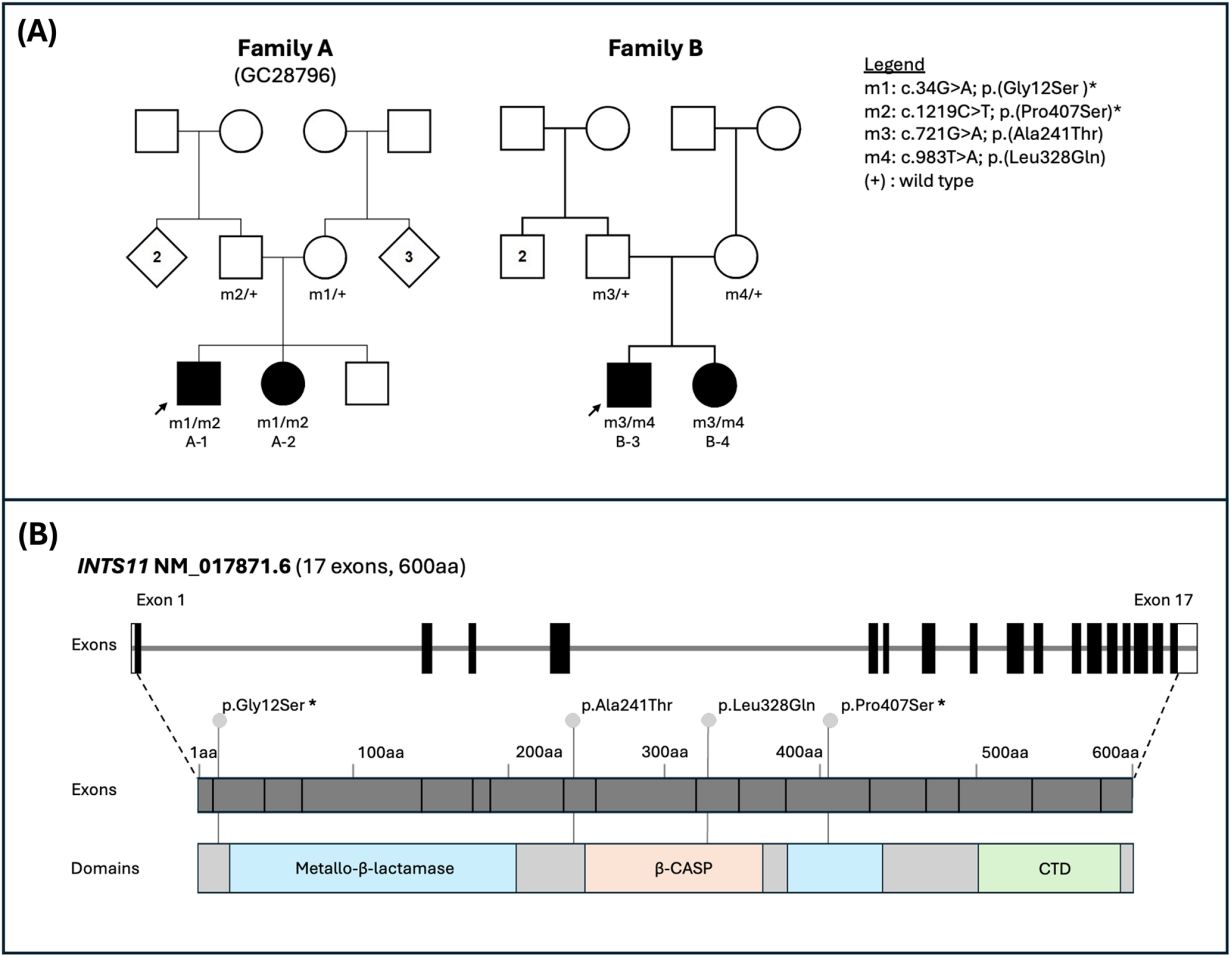
**Family pedigrees and *INTS11* variant localization.** (**A**) Family pedigrees for family A (individuals A-1 and A-2) and family B (individuals B-3 and B-4) showing genotypes and familial segregation. Previously reported variants are indicated with an *asterisk* (*) within the legend. (**B**) Genomic and protein domain organization of human *INTS11* (NM_017871.6), which comprises 17 exons and encodes for a 600–amino acid (aa) protein containing the following domains: metallo-β-lactamase domain (*light blue*), β-CASP (*amber*), and C-terminal domain (CTD) (*green*). Variants identified in this study are mapped to their respective exon and protein domain locations in the diagram; previously reported variants are marked with an *asterisk* (*).

**Figure 2.**
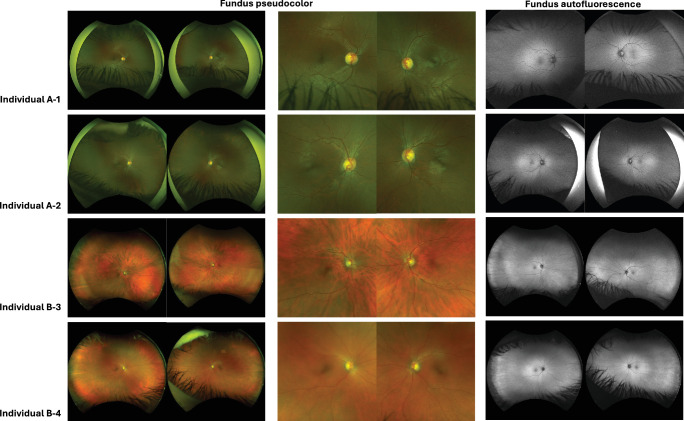
**Ultra-widefield pseudocolor and autofluorescence fundus imaging for individuals A-1, A-2, B-3, and B-4.** Mild disc pallor is noted for all four individuals, but the pseudocolor and autofluorescence imaging are otherwise unremarkable.

The pedigrees of the two families are shown in Figure 1. Clinical features are presented first (Figs. 2^3^,^4^–5), followed by the genetic findings (variants are also shown in Fig. 1). Table 1 summarizes the clinical and genetic details for all four affected individuals.

**Figure 3.**
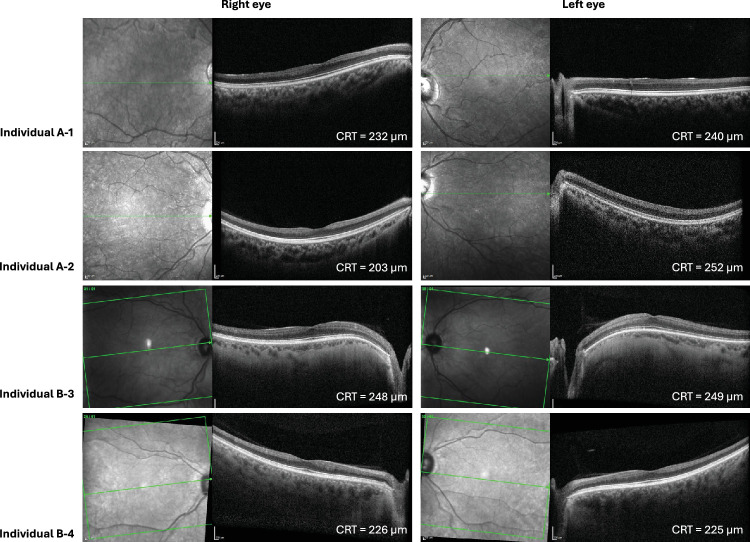
**SD-OCT imaging for individuals A-1, A-2, B-3, and B-4.** OCT scans are notable for marked inner retinal thinning with preserved outer retinal layers in all four individuals.

**Figure 4.**
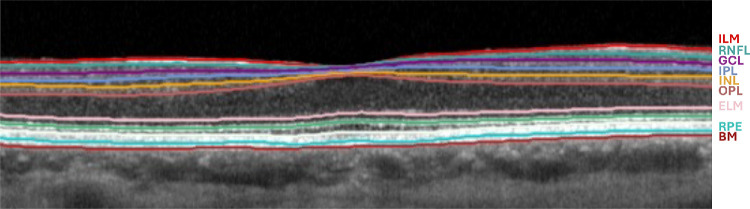
**Macular OCT segmentation of the right eye in individual B-3.** Automated macular segmentation of SD-OCT of the right eye of individual B-3 at age 48 years, performed using the built-in Heidelberg Eye Explorer software (Heidelberg Engineering, Heidelberg, Germany). This was selected as a representative example due to the availability of high-quality volumetric scans suitable for automated segmentation. The segmentation demonstrates marked thinning of inner retinal layers, including the retinal nerve fiber layer (RNFL), ganglion cell layer (GCL), inner plexiform layer (IPL), and inner nuclear layer (INL), with relative preservation of outer retinal layers, including the outer nuclear layer (ONL) and retinal pigment epithelium (RPE). The internal limiting membrane (ILM), external limiting membrane (ELM), and Bruch's membrane (BM) are also indicated.

**Figure 5.**
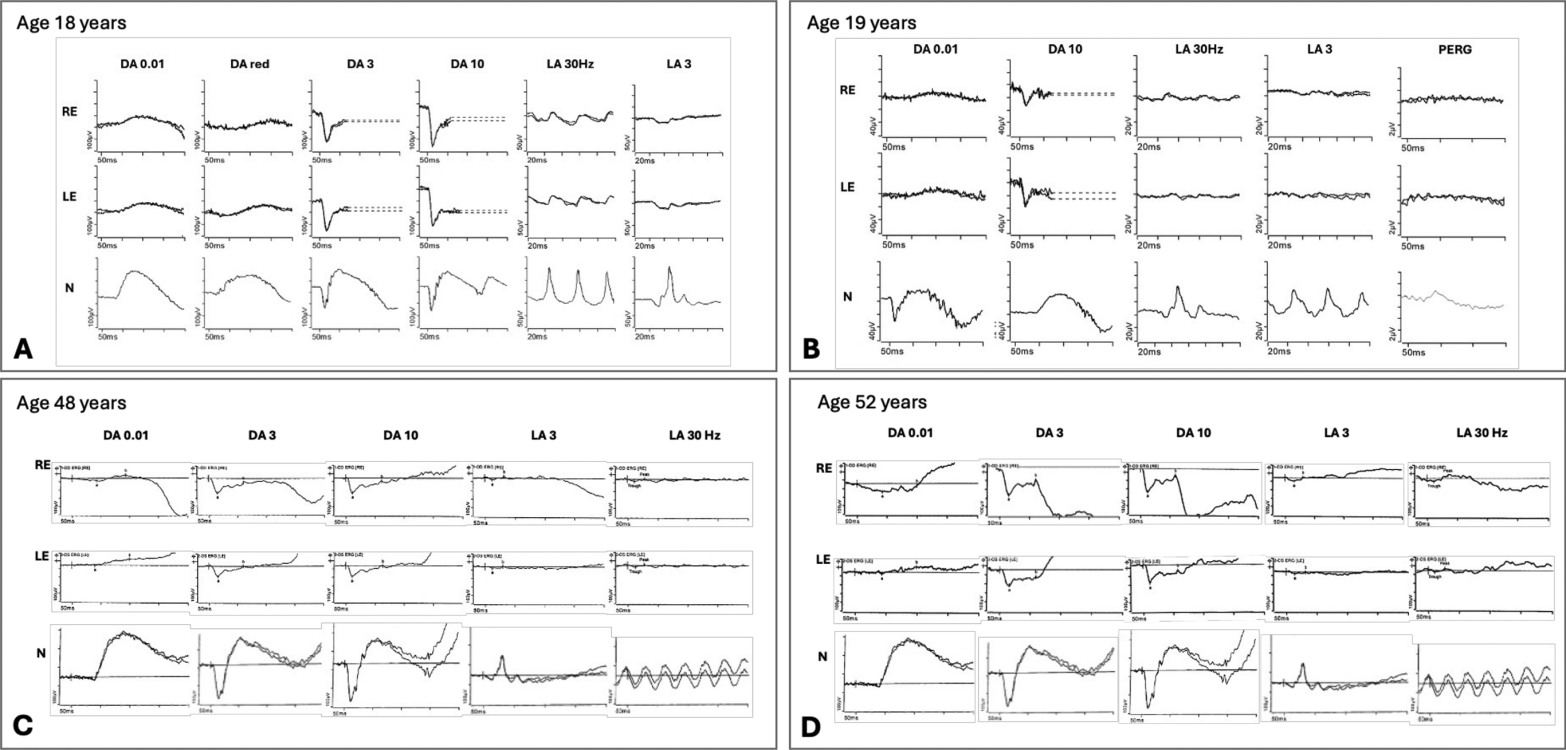
**Electroretinography findings for individuals A-1, A-2, B-3, and B-4.** Full-field ERGs from the right eye (RE) and left eye (LE) of individuals A-1, A-2, B-3, and B-4 (**A–D**, respectively). Individual A-1 (**A**) was tested according to ISCEV Standard methods incorporating an additional DA red flash ERG, using silver thread corneal electrodes. Individual A-2 (**B**) was tested according to the ISCEV-recommended abbreviated protocol (dark adaptation time 10 minutes; no mydriasis), using lower eyelid skin electrodes; pattern ERGs (PERGs) were also recorded with skin electrodes. *Traces* from a representative control subject are shown for comparison (N), *waveforms* are superimposed to demonstrate reproducibility, and *broken lines* replace blink and eye movement artifacts (**A**, **B**). Individual B-3 (**C**) and individual B-4 (**D**) were tested according to ISCEV Standard methods using silver thread corneal electrodes to include the DA 0.01, DA 3, DA 10, LA 3, and LA 30-Hz ERGs. Traces from a representative control subject are shown for comparison (N). (**A**) Full-field ERGs from the RE and LE of individual A-1 at age 18 years. The DA 0.01 is subnormal, and the DA red flash ERG lacks the cone-mediated x-wave with only relative preservation of the rod mediated b-wave. The DA 3 and DA 10 ERGs have preserved a-waves but electronegative waveforms (b/a ratios <1). The LA 30-Hz ERG is mildly delayed and of subnormal amplitude, and LA 3 ERGs show a broadened waveform with a- and b- wave reduction and a low b/a ratio. PERGs were not recordable due to the effects of nystagmus. (**B**) Full-field ERGs from the RE and LE of individual A-2 at age 19 years. DA 0.01 ERGs are subnormal and DA 10 ERGs electronegative, with preserved a-waves. LA 3 ERGs are subnormal with a low b/a ratio, and LA 30-Hz ERGs are delayed and subnormal. Pattern ERG P50 components are undetectable, suggesting macular involvement. (**C**) Full-field ERGs from the RE and LE of individual B-3 at age 48 years. The DA 0.01 is subnormal. DA 3 and DA 10 ERGs are electronegative with preserved a-waves (b/a ratios <1) and delayed b-waves. The LA 3 ERGs show reduced b-wave. The LA 30-Hz ERG is of subnormal amplitude. (**D**) Full-field ERGs from the RE and LE of individual B-4 at age 52 years. The DA 0.01 ERGs appear within reference levels. The DA 3 and DA 10 ERGs show delayed b-waves with electronegative waveforms (b/a ratio <1). The LA 3 ERGs show mildly reduced b-wave in RE and reduced and delayed b-wave in LE. The LA 30-Hz ERGs appear reduced.

**Table 1.** Clinical and Genetic Findings for Individuals A-1, A-2, B-3, and B-4 With Biallelic *INTS11* Variants (NM_017871.6)

	A (GC28796)		B	
FamilyIndividual	A-1	A-2	B-3	B-4
Allele 1	c.34G> A; p.(Gly12Ser)	c.34G > A; p.(Gly12Ser)	c.721G > A; p.(Ala241Thr)	c.721G > A; p.(Ala241Thr)
Allele 2	c.1219C > T; p.(Pro407Ser)	c.1219C > T; p.(Pro407Ser)	c.983T > A; p.(Leu328Gln)	c.983T > A; p.(Leu328Gln)
Country of recruitment	UK	UK	USA	USA
Ethnicity	Asian Bangladeshi	Asian Bangladeshi	Caucasian	Caucasian
Sex	Male	Female	Male	Female
Age at last examination	22 years	20 years	48 years	51 years
Ocular features				
BCVA (Snellen)	OD: 20/40	OD: 20/60	OD: 20/30	OD: 20/30
	OS: 20/30	OS: 20/50	OS: 20/40	OS: 20/30
Refraction	OD: +3.25/−1.00 × 90°	OD: +1.00/−0.75 × 165°	OD: −6.00/−1.00 × 10°	OD: −1.75/−2.00 × 115°
	OS: +3.00/−1.00 × 55°	OS: +0.75/−0.75 × 10°	OS: −6.25/−0.75 × 118°	OS: −2.75/−1.25 × 76°
Anterior segment	Within normal limits	Within normal limits	Within normal limits	1+ nuclear sclerosis cataract in both eyes
Fundus	Mild optic disc pallor, no atrophic or pigmentary retinal changes	Mild optic disc pallor, no atrophic or pigmentary retinal changes	Optic disc pallor, no atrophic or pigmentary retinal changes	Optic disc pallor, no atrophic or pigmentary retinal changes
AF	No AF changes of note	No AF changes of note	No AF changes of note	No AF changes of note
OCT	Severe loss of inner retinal layers, outer retinal layers appear intact	Severe loss of inner retinal layers, outer retinal layers appear intact	Severe loss of inner retinal layers, outer retinal layers appear intact	Severe loss of inner retinal layers, outer retinal layers appear intact
ERG (age of examination)	Electronegative ERG (18 years)	Electronegative ERG (19 years)	Electronegative ERG (42 years)	Electronegative ERG (45 years)
Other ocular features	Periodic alternating nystagmus	Periodic alternating nystagmus	Bilateral ptosis, glaucoma suspect	None
Systemic findings				
Neurologic findings	Global developmental delay, motor delay with ataxia, cognitive impairment with limited speech, hypotonia, microcephaly, epilepsy	Global developmental delay, motor delay with ataxia, cognitive impairment with limited speech, hypotonia	Mild intellectual disability, autism spectrum disorder; head circumference within normal limits (no microcephaly)	Severe intellectual disability, gross motor and speech delay, severely spastic lower extremities, autism spectrum disorder; head circumference within normal limits (no microcephaly)
Brain MRI (age of examination)	Small cerebellum; delayed myelination (2 years)	Progressive cerebellar atrophy; milder pontine volume loss; diffuse white matter signal changes (9 years)	Optic nerve thinning, no other structural abnormalities (47 years)	No morphologic abnormalities of the brain parenchyma (50 years)
Other systemic findings	—	—	Type 2 diabetes, kidney stones, abdominal hernia, obstructive sleep apnea	Scoliosis, kyphosis, thyroid disease, obstructive sleep apnea

AF, autofluorescence; OD, right eye; OS, left eye.

### Clinical Findings in Family A

Individual A-1 (22-year-old man) and individual A-2 (21-year-old woman) are siblings born to unrelated, unaffected parents of Asian Bangladeshi ancestry. Both have a shared systemic phenotype with clinical features, including developmental motor delay with ataxia, cognitive impairment with limited speech, microcephaly, epilepsy, and periodic alternating nystagmus, as previously described.^8^

On ophthalmic examination, individual A-1 achieved Snellen best-corrected visual acuities (BCVAs) of 20/40 in the right eye and 20/30 in the left eye, while individual A-2 achieved BCVAs of 20/60 in the right eye and 20/50 in the left eye. Both had a low hypermetropic prescription (individual A-1: right eye +3.25/−1.00 × 90°; left eye +3.00/−1.00 × 55°; individual A-2: right eye +1.00/−0.75 × 165°; left eye +0.75/−0.75 × 10°). Anterior segment examination was unremarkable for both siblings. Fundus examination revealed mild optic disc pallor, but no obvious additional retinal abnormalities were observed on ultra-widefield pseudocolor or autofluorescence imaging; fundus images are shown in Figure 2. SD-OCT scans demonstrated a distinctive retinopathy characterized by severe thinning of inner retinal layers, including the ganglion cell layer and inner nuclear layer, while the outer retinal layers (including the outer nuclear layer) appeared intact (Fig. 3). Full-field ERG abnormalities in individuals A-1 and A-2 (Figs. 5A, 5B) included electronegative DA strong flash ERGs (DA3 and/or DA10) waveforms, consistent with generalized rod and cone system dysfunction with a locus postphototransduction or inner retinal bilaterally. Pattern ERGs, available in individual A-2, revealed undetectable P50 components, in keeping with macular involvement bilaterally.^13^^,^^19^^–^^21^

### Clinical Findings in Family B

Individual B-3 (48-year-old man) and individual B-4 (51-year-old woman) are siblings born to unrelated, unaffected parents.

Individual B-3 was diagnosed with autism spectrum disorder in childhood and had mild intellectual disability requiring special education services throughout schooling. He began noticing visual issues at ages 40 to 41 years, including night blindness and blurred vision. Ophthalmic history was also significant for bilateral ptosis and suspected glaucoma on the basis of retinal nerve fiber layer changes and a paternal history of glaucoma. He achieved Snellen BCVAs of 20/30 in the right eye and 20/40 in the left eye with a myopic prescription (right eye −6.00/−1.00 × 10°, left eye −6.25/−0.75 × 118°). Neurology assessment was notable for mild cognitive disorder with a Montreal Cognitive Assessment (MoCA) score of 18 and difficulties with remote memory. Head circumference measured in adulthood was within normal limits, with no evidence of microcephaly. Neuroimaging (magnetic resonance imaging [MRI]) did not show any structural abnormalities other than optic nerve thinning.

Individual B-4 was diagnosed with developmental delay in childhood, including gross motor delay (ambulation began at age 4 years) and speech delay (began at age 7–8 years). She was also diagnosed with autism spectrum disorder and cerebral palsy in childhood, with severely spastic lower extremities. She has severe intellectual disability, requiring special education throughout school, with a MoCA score of 8. Adult head circumference was within the normal range, and microcephaly was not present. MRI showed no morphologic abnormalities of the brain parenchyma. On ophthalmic examination, she achieved a Snellen BCVA of 20/30 in both eyes with a myopic prescription (right eye −1.75/−2.00 × 115°, left eye −2.75/−1.25 × 76°).

Dilated fundus examination and pseudocolor imaging for both individuals B-3 and B-4 showed bilateral optic disc pallor (Fig. 2), and OCT scans showed inner retinal layer thinning (Figs. 3, 4; Supplementary Fig. S1, and Supplementary Figure S2). In individuals B-3 and B-4, longitudinal OCT imaging demonstrated relative structural stability over time; representative longitudinal segmentation of the right eye of individual B-3 over a 7-year interval is shown in Supplementary Figure S1 and Supplementary Table S1. Electroretinography in individuals B-3 and B-4 showed electronegative DA 3 and DA 10 waveforms, with reduced b-waves on LA-3 testing, consistent with inner retinal rod and cone system dysfunction (Figs. 5C, 5D).

### Genetic Findings

In individuals A-1 and A-2, genomic analysis identified biallelic missense *INTS11* variants (NM_017871.6) c.34G > A; p.(Gly12Ser) and c.1219C > T; p.(Pro407Ser), as previously described^8^ (Table 2). In individuals B-3 and B-4, novel biallelic missense *INTS11* variants were identified: c.721G > A; p.(Ala241Thr) and c.983T > A; p.(Leu328Gln). All four variants were either absent from the gnomAD (v4.1.0) population database or observed at extremely low allele frequencies, with no homozygous individuals reported. In silico predictions of these variants are supportive of pathogenicity, and their details are summarized in Table 2.

**Table 2.** *INTS11* Variants (NM_017871.6) Identified in Families A and B in This Study

						In Silico Predictions^†^				
Family	Variant (Nucleotide)	Variant (Protein)	Location	Variant Type	gnomAD MAF^*^	Revel	AlphaMissense	SpliceAI	ClinVar (ID)	Published
A	c.34G > A	p.(Gly12Ser)	Exon 2	Missense	0.000003105	Uncertain (0.64)	Deleterious (0.969)	Benign (0.04)	Absent	Tepe et al.^8^
B	c.721G > A	p.(Ala241Thr)	Exon 8	Missense	0.00005660	Uncertain (0.63)	Deleterious (0.989)	Benign (0.01)	VUS (2580096)	Novel
B	c.983T > A	p.(Leu328Gln)	Exon 10	Missense	Absent	Deleterious (0.78)	Deleterious (0.999)	Benign (0.02)	VUS (2580101)	Novel
A	c.1219C > T	p.(Pro407Ser)	Exon 12	Missense	0.000001240	Deleterious (0.69)	Deleterious (0.948)	Benign (0)	Absent	Tepe et al.^8^

MAF, minor allele frequency; VUS, variant of uncertain significance.

* gnomAD, Genome Aggregation Database v4.1.0. No homozygous individuals were identified for each of the identified variants in gnomAD.

† REVEL is an ensemble score based on 13 individual scores for predicting the pathogenicity of missense variants. AlphaMissense scores can be interpreted as the approximate probability of a variant being clinically pathogenic. SpliceAI delta scores can be interpreted as the probability that the variant affects splicing at any position within a ±500-bp window around it. Scores for REVEL, AlphaMissense, and SpliceAI range from 0 to 1, with higher scores indicating a higher probability of the variant being damaging or having a splice-altering effect.

Parental segregation analysis confirmed that the *INTS11* variants in affected individuals in both families were in *trans*, consistent with an autosomal recessive inheritance pattern (Fig. 1). Although all four variants are currently classified as variants of uncertain significance,^17^^,^^18^ the strong neurodevelopmental phenotypic overlap with previously reported cases of *INTS11*-associated neurodevelopmental disorder,^8^^,^^22^^,^^23^ along with the shared clinical features across affected individuals in both families, supports the interpretation of these variants as the likely molecular diagnoses.

Individuals A-1 and A-2 had no other plausible pathogenic genotypes in known disease-associated genes identified by the analysis pipeline. Individual B-4, however, was noted to carry a maternally inherited heterozygous *AUTS2* variant (NM_015570.4): c.2119C > T; p.(Arg707Trp). *AUTS2* encodes a nuclear protein involved in transcriptional regulation, and pathogenic loss-of-function variants have been linked to an autosomal dominant neurodevelopmental disorder characterized by variable intellectual disability, developmental delay, microcephaly, hypotonia, and autistic traits.^24^^–^^29^ The *AUTS2* p.(Arg707Trp) variant has been observed at low frequency in population cohorts (six heterozygous carriers in gnomAD v4.1.0), and in silico predictions suggest a possible deleterious effect (REVEL score 0.3; AlphaMissense score 0.871; SpliceAI score 0.23). Notably, individual B-3, who exhibits a highly similar neurodevelopmental presentation, does not carry the variant, while the unaffected mother does carry the variant. The clinical significance of this variant therefore remains uncertain. Importantly, retinal abnormalities have not been reported in association with *AUTS2*-associated neurodevelopmental disease to date.

## Discussion

Biallelic variants in *INTS11* were first described as a cause of human disease by Tepe et al.,^8^ who reported 15 individuals from 10 unrelated families with a neurodevelopmental disorder characterized by global developmental and language delay, intellectual disability, and impaired motor development. This was subsequently followed by two additional case reports: Kuang et al.^23^ described two Chinese siblings with a homozygous missense *INTS11* variant, while Jiang et al.^22^ reported a 2-year-old Chinese girl with compound heterozygous *INTS11* variants. All reported cases demonstrated overlapping neurodevelopmental features, further supporting an emerging recessive *INTS11*-associated neurodevelopmental syndrome.

Retinal dystrophy was previously noted in two siblings with *INTS11*-associated neurodevelopmental disorder^8^—corresponding to individuals A-1 and A-2 in this study—but the retinal phenotype was not characterized, and no retinal imaging or electrophysiology findings were provided. In this study, we present the first detailed assessment of their retinal phenotype, demonstrating a distinctive pattern of marked inner retinal thinning with preservation of the outer retina (Figs. 3, 4; Supplementary Table S1) and an electronegative ERG consistent with inner retinal dysfunction (Fig. 5). The identification of two additional individuals (individuals B-3 and B-4) with similar retinal findings consolidates retinopathy as a feature of *INTS11*-associated neurodevelopmental disorder and suggests that the retinal involvement follows a characteristic pattern, thereby better defining the phenotypic spectrum of this newly described condition.

Longitudinal structural data in one representative individual (Supplementary Fig. S1; Supplementary Table S1) demonstrate minimal change in retinal layer thickness over a 7-year interval; similar qualitative findings were observed in the fellow eye of this individual and in both eyes of individual B-4. While the number of individuals with longitudinal data remains small, the relative stability observed in these cases suggests that the retinal changes may be established early in life and remain largely nonprogressive over mid-adulthood. Longer-term follow-up across additional affected individuals will be required to determine whether subtle progression occurs and to more fully define the natural history of the retinal phenotype in *INTS11*-associated disease.

Additional ocular features reported in individuals with *INTS11*-associated neurodevelopmental disorder, including those in the current case series, include optic atrophy or pallor (9/20), nystagmus (5/20), refractive error (myopia, hypermetropia, or astigmatism; 5/20), strabismus (2/20), and bilateral ptosis in one individual. In our cohort, all four affected individuals demonstrated mild optic disc pallor and two had nystagmus, features that were also noted in several reported cases.

The inner retinal phenotype we describe is subtle on ophthalmoscopy and may be difficult to detect and interpret without specialist ophthalmic assessment and investigations such as OCT and electrophysiology (Figs. 3^4^–5). Here, we present quantitative macular OCT analysis (Supplementary Table S1), demonstrating thinning across multiple retinal layers, with disproportionate involvement of the inner retinal layers, including the ganglion cell, inner plexiform, and inner nuclear layers. Consistent with these structural findings, the presence of an electronegative ERG—typically associated with dysfunction at the photoreceptor–bipolar cell synapse or within the inner retina rather than primary optic nerve pathology—supports inner retinal dysfunction. In this context, the clinically pale optic discs reflect a wider loss of inner retina that involves more than the ganglion cell and nerve fiber layers, rather than selective optic atrophy. It is therefore plausible that similar inner retinal involvement was present but not recognized in the additional individuals previously reported, particularly as detailed retinal imaging was not performed in those cases; in the presence of a pale optic disc, visual impairment in affected individuals could reasonably have been misattributed to optic atrophy instead. It also remains possible that the retinal phenotype associated with *INTS11* dysfunction is broader than currently described and may extend beyond the inner retina to include involvement of the outer retina, as additional individuals are identified and undergo detailed retinal phenotyping. Recognizing retinopathy as part of the *INTS11* phenotypic spectrum underscores the importance of comprehensive ophthalmologic evaluations in affected individuals, allowing for early recognition of visual impairment and provision of appropriate support.

Variants in other Integrator subunits, including *INTS1*, *INTS8*, and *INTS13*, have also been linked to recessive neurodevelopmental disorders with associated ocular anomalies. Biallelic *INTS1* variants are associated with childhood-onset cataracts, microphthalmia, microcornea, and iris and retinal colobomas,^10^^,^^11^ *INTS8* variants have been linked to recessive optic atrophy^11^ and *INTS13* variants with an oral-facial-digital-like syndrome with nonspecific ophthalmic findings, including dilated retinal vessels and a crowded optic disc. Additionally, a dominant variant in *INTS15* has been implicated in a spectrum of ocular malformations, including corneal opacity, microcornea, iris anomaly, cataract, persistent fetal vasculature, dislocation and hypoplasia of the fovea, myelinated retinal nerve fibers, retinal degeneration, atrophy of the retina and choroid, and optic nerve anomalies.^9^ These findings support a role for the Integrator complex in ocular and retinal development.

Loss of *Ints11* in *Drosophila* and mice leads to embryonic lethality^23^^,^^30^; however, the effect of *INTS11* dysfunction in ocular models has not been assessed. Future studies investigating the role of *INTS11* in ocular and retinal development and function could provide valuable mechanistic insights.

The Integrator complex plays a critical role in snRNA processing, and variants in snRNA genes have also been linked to neurodevelopmental disorders with retinal phenotypes. *RNU4atac* variants cause recessive Roifman and Lowry–Wood syndrome with associated retinal dystrophy.^31^ Recurrent de novo variants in the U4 snRNA gene *RNU4-2* were recently discovered to cause neurodevelopmental disease^32^^,^^33^; variants in *RNU4-2* and in four of five *RNU6* paralogues have also recently been associated with nonsyndromic retinitis pigmentosa.^34^ The retinal dystrophy in these conditions, however, is typically characterized by progressive rod-cone dystrophy, which contrasts with presumed congenital and stable inner retinal maldevelopment observed in *INTS11*-associated disease.

*WDR73*, an *INTS11* interactor, is implicated in Galloway–Mowatt syndrome, a rare autosomal recessive condition with central nervous system, renal, and ocular abnormalities.^35^ The retinopathy described in individuals with Galloway–Mowatt syndrome has been associated with an abnormal photopic ERG (no scotopic ERGs performed), and another case report has described residual ISCEV Standard LA ERGs and DA strong flash ERG a-wave reductions, but with a low b/a ratio or more severely reduced b-wave, giving rise to a negative waveform.^36^^–^^38^ The latter potentially indicates shared pathways of retinal maldevelopment (although a negative DA ERG waveform in the presence of a subnormal a-wave can reflect a relatively preserved dark-adapted cone system response).^39^^,^^40^

Most monogenic retinopathies affect the outer retina, evident both structurally on retinal imaging and functionally on ERG testing. Electronegative ERGs (with normal amplitude a-waves) are seen in some inherited retinal disorders, but usually without inner retinal layer loss on imaging. Such conditions include the “complete” and “incomplete” forms of congenital stationary night blindness, X-linked retinoschisis, and *CRX*-associated disease.^41^ However, the retinal phenotype observed in *INTS11*-associated disease, characterized by inner retinal thinning and preserved outer retinal layers, is distinct and not shared with these conditions.

Although other inherited retinal diseases with both inner retinal thinning and an electronegative ERG have not been reported, certain acquired conditions may demonstrate either or both features. These include retinovascular occlusions, quinine toxicity, and some cases of melanoma-associated retinopathy.^41^^–^^43^ In Alport syndrome, inner retinal thinning can be observed, but this is usually confined to the macular area and frequently associated with an irregular macular profile.^44^ Inner retinal dysfunction has been reported in association with heterozygous variants in *ITM2B*, but OCT images in those cases show hyperreflectivity in the inner retinal layers.^45^^,^^46^

*INTS11*-associated neurodevelopmental syndrome is a newly described disorder, with only 18 affected individuals reported to date, including individuals A-1 and A-2 described in this study. Here, we describe two novel variants in two additional affected individuals, expanding the genotypic and phenotypic spectrum of *INTS11*-associated disorders. Detailed characterization of the retinal phenotype is provided in four affected individuals, consolidating a distinctive retinopathy as a feature of *INTS11*-associated disease.

Neurodevelopmental disorders are genetically heterogeneous and challenging to diagnose. Despite recent advancements in genomic technologies, the diagnostic yield remains low.^28^ Recognizing distinctive phenotypes, such as the unique pattern of presumed inner retinal maldevelopment observed in some cases of *INTS11*-associated neurodevelopmental disorders, can provide valuable diagnostic clues facilitating the identification of pathogenic variants and an accurate molecular diagnosis, with implications for family counseling and clinical care.

## Supplementary Material

Supplement 1
