# Supplementary material for: Characterizing a Unique Retinal Phenotype in INTS11-Associated Neurodevelopmental Disorder
Source: Invest Ophthalmol Vis Sci. 2026 Mar 11;67(3):21. doi: 10.1167/iovs.67.3.21 (PMC12988679; doi:10.1167/iovs.67.3.21)
Supplement: Supplement 1 [file iovs-67-3-21_s001.docx]

**SUPPLEMENTAL INFORMATION**

**Supplemental Figure S1. Longitudinal macular OCT segmentation in Individual B-3 at ages 41 and 48 years**

**
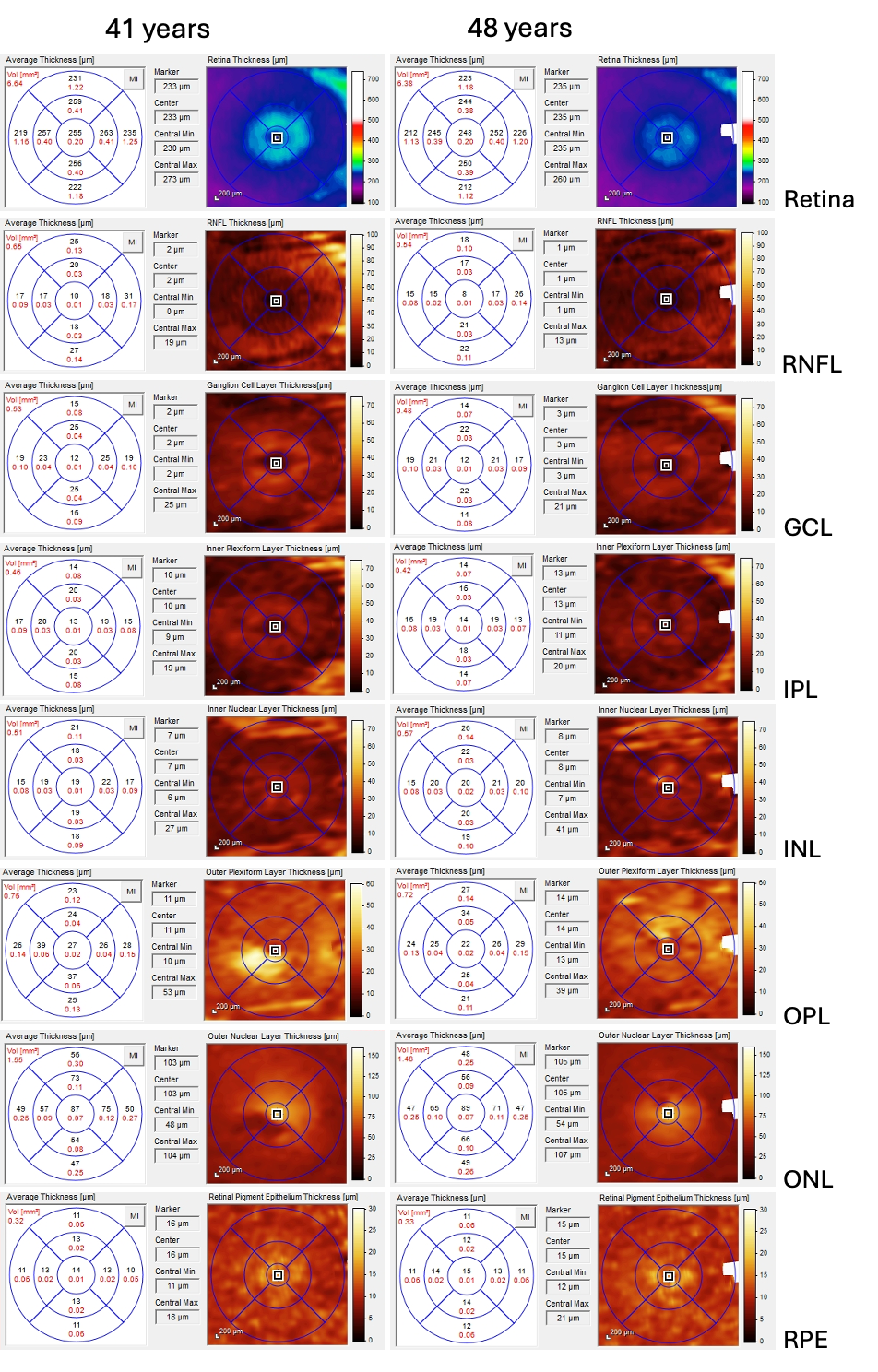
**

Supplemental Figure S1. Automated macular segmentation of spectral-domain optical coherence tomography (SD-OCT) of the right eye of Individual B-3 performed at ages 41 years (left column) and 48 years (right column) using the Heidelberg Eye Explorer software (Heidelberg Engineering, Heidelberg, Germany). This individual was chosen as a representative example due to the availability of high-quality scans suitable for automated segmentation at both time points. Thickness maps and Early Treatment Diabetic Retinopathy Study (ETDRS) subfield values are shown for the full retina and individual retinal layers, including the retinal nerve fiber layer (RNFL), ganglion cell layer (GCL), inner plexiform layer (IPL), inner nuclear layer (INL), outer plexiform layer (OPL), outer nuclear layer (ONL), and retinal pigment epithelium (RPE). Comparison across the two time points demonstrates minimal change over the 7-year interval, supporting relative longitudinal stability of the retinal phenotype.

**Supplemental Figure S2. Longitudinal optic disc OCT imaging in individual B-3**


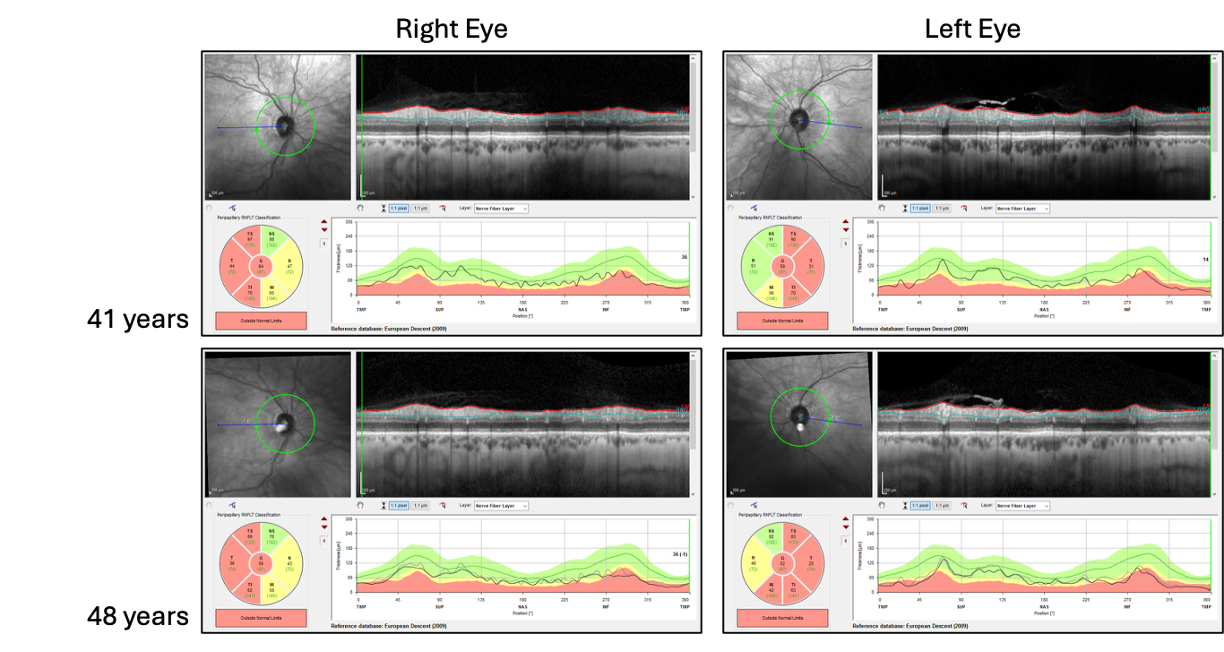


Supplemental Figure S2. Optic disc retinal nerve fibre layer (RNFL) analysis obtained using Spectralis spectral-domain optical coherence tomography (Heidelberg Engineering, Heidelberg, Germany) from individual B-3, showing right and left eyes imaged at ages 41 years (top row) and 48 years (bottom row). For each eye and time point, the peripapillary infrared fundus image with scan location, corresponding B-scan, and RNFL thickness profile are shown relative to the device reference database. These longitudinal images demonstrate predominantly temporal RNFL thinning in both eyes, with no clear evidence of substantial interval change over the seven-year follow-up period, indicating relative stability of peripapillary RNFL structure.

**Supplemental Table S1. Longitudinal and retinal layer-specific macular OCT thickness measurements in Individual B-3 compared with published reference values**

| **Layer** | **ETDRS Subfield** | **Published reference dataset^a^, mean ± SD (μm)** | **Thickness at age 41 years (μm)** | **Thickness at age 48 years (μm)** | **Change (41 → 48 years) (μm)** | **Difference (48 years) vs published reference (z-score)** |
| --- | --- | --- | --- | --- | --- | --- |
| Full retina | Center | 280.1 ± 17.5 | 255.0 | 248.0 | -7.0 | -1.8 SD |
|  | Middle ring | 343.4 ± 13.9 | 258.8 | 247.8 | -11.0 | -6.9 SD |
|  | Outer ring | 298.2 ± 12.8 | 226.8 | 218.3 | -8.5 | -6.2 SD |
| RNFL | Center | 12.8 ± 1.8 | 10.0 | 8.0 | -2.0 | -2.7 SD |
|  | Middle ring | 23.2 ± 1.7 | 18.3 | 17.5 | -0.8 | -3.4 SD |
|  | Outer ring | 38.8 ± 4.4 | 25.0 | 20.3 | -4.7 | -4.2 SD |
| GCL | Center | 17.2 ± 3.9 | 12.0 | 12.0 | 0 | -1.3 SD |
|  | Middle ring | 52.7 ± 4.0 | 24.5 | 21.5 | -3.0 | -7.8 SD |
|  | Outer ring | 35.4 ± 3.1 | 17.3 | 16.0 | -1.3 | -6.3 SD |
| IPL | Center | 22.5 ± 3.2 | 13.0 | 14.0 | +1.0 | -2.7 SD |
|  | Middle ring | 42.4 ± 2.8 | 19.8 | 18.0 | -1.8 | -8.7 SD |
|  | Outer ring | 28.9 ± 2.3 | 15.3 | 14.3 | -1.0 | -6.3 SD |
| INL | Center | 19.4 ± 4.5 | 19.0 | 20.0 | 1.0 | +0.1 SD |
|  | Middle ring | 39.7 ± 3.0 | 19.5 | 20.8 | 1.3 | -6.3 SD |
|  | Outer ring | 32.6 ± 2.2 | 17.8 | 20.0 | 2.2 | -5.7 SD |
| OPL | Center | 26.0 ± 6.0 | 28.0 | 22.0 | -6.0 | -0.7 SD |
|  | Middle ring | 33.1 ± 4.5 | 31.5 | 27.5 | -4.0 | -1.2 SD |
|  | Outer ring | 26.9 ± 2.2 | 25.5 | 25.3 | -0.2 | -0.7 SD |
| ONL | Center | 92.2 ± 9.3 | 87.0 | 89.0 | 2.0 | -0.3 SD |
|  | Middle ring | 71.0 ± 7.6 | 64.8 | 64.5 | -0.3 | -0.9 SD |
|  | Outer ring | 57.7 ± 6.0 | 50.5 | 47.8 | -2.7 | -1.7 SD |
| RPE | Center | 17.4 ± 1.9 | 14.0 | 15.0 | 1.0 | -1.3 SD |
|  | Middle ring | 15.1 ± 1.4 | 13.0 | 13.3 | -0.3 | -1.3 SD |
|  | Outer ring | 13.3 ± 1.3 | 10.8 | 11.0 | 0.2 | -1.8 SD |

Supplementary Table S3. Quantitative macular optical coherence tomography (OCT) thickness measurements for the right eye of Individual B-3, assessed at ages 41 and 48 years, with comparison to a published reference dataset. This individual was selected as a representative example due to the availability of high-quality volumetric scans suitable for automated segmentation at both time points. Thickness values are shown for the full retina and individual retinal layers across Early Treatment Diabetic Retinopathy Study (ETDRS) subfields (central 1 mm, inner ring 1–3 mm, outer ring 3–6 mm).

^a^Reference macular thickness values are derived from Invernizzi et al.^1^, a large spectral-domain OCT study of healthy White individuals aged 20–74 years, and were used to provide an age-inclusive reference comparison in the absence of device-specific age-matched controls. Reference values are presented as mean ± standard deviation (SD).

Longitudinal change represents the absolute difference in thickness between ages 41 and 48 years. The difference between the age 48 measurement and the published reference dataset is expressed as a z-score, calculated as: (observed thickness − reference mean) / reference SD. Negative z-scores indicate thinning relative to the reference population.

This analysis demonstrates marked thinning of inner retinal layers, including the retinal nerve fiber layer (RNFL), ganglion cell layer (GCL), inner plexiform layer (IPL), and inner nuclear layer (INL), often exceeding −5 SD relative to reference values. In contrast, outer retinal layers, including the outer nuclear layer (ONL) and retinal pigment epithelium (RPE), show relative preservation, with z-scores generally within or close to the reference range. Despite deviation from reference values, longitudinal measurements over the 7-year interval show minimal change, supporting relative stability of the retinal phenotype.

Abbreviations: RNFL, retinal nerve fiber layer; GCL, ganglion cell layer; IPL, inner plexiform layer; INL, inner nuclear layer; OPL, outer plexiform layer; ONL, outer nuclear layer; RPE, retinal pigment epithelium; ETDRS, Early Treatment Diabetic Retinopathy Study.

**References**

1. Invernizzi A, Pellegrini M, Acquistapace A, et al. Normative Data for Retinal-Layer Thickness Maps Generated by Spectral-Domain OCT in a White Population. *Ophthalmol Retina*. Aug 2018;2(8):808-815 e801. doi:10.1016/j.oret.2017.12.012
